# DUSP1 Attenuates Renal Injury in Diabetic Nephropathy by Modulating Ferroptosis: Evidence From Animal Experiments

**DOI:** 10.1002/iid3.70340

**Published:** 2026-02-04

**Authors:** Jiarong Liu, Junping Zhang, Yun Zou, Wen Chen, Jixiong Xu

**Affiliations:** ^1^ Department of Endocrine and Metabolism, The First Affiliated Hospital, Jiangxi Medical College Nanchang University Nanchang Jiangxi China; ^2^ Jiangxi Clinical Research Center for Endocrine and Metabolic Disease Nanchang Jiangxi China; ^3^ Jiangxi Branch of National Clinical Research Center for Metabolic Disease Nanchang Jiangxi China

**Keywords:** animal experiments, diabetic nephropathy, DUSP1, ferroptosis

## Abstract

**Objective:**

Emerging evidence suggests that ferroptosis contributes significantly to the progression of diabetic nephropathy (DN). This study aimed to explore the potential association between dual specificity phosphatase 1 (DUSP1) and ferroptosis in a streptozotocin‐induced DN rat model.

**Methods:**

We analyzed microarray datasets (GSE30122 and GSE96804) from the gene expression omnibus (GEO) database to identify ferroptosis‐related differentially expressed genes (FDEGs), with particular focus on DUSP1. Experimental validation was performed using 45 specific pathogen‐free Sprague‐Dawley rats: 15 controls and 30 STZ‐induced DN models (60 mg/kg, i.p.). After 12 weeks, successfully modeled rats (*n* = 28) were randomized into DN (*n* = 14) and DN+Ferrostatin‐1 (Fer‐1, a ferroptosis inhibitor, 2.5 μmol/kg, *n* = 14) groups. Renal function parameters (blood urea nitrogen, serum creatinine, urinary albumin) were quantified using automated biochemical analysis. Renal tissue antioxidant capacity (SOD, GSH, MDA) and iron content were assessed. Histopathological evaluation employed HE, Masson, PAS, and Lillie staining. DUSP1 expression was analyzed via immunohistochemistry, Western blot, and RT‐qPCR.

**Results:**

DN rats exhibited characteristic metabolic disturbances including polydipsia (394.32 ± 9.92 vs. 28.84 ± 2.45 mL/day, *p* < 0.001), polyuria, and progressive weight loss. Renal function impairment was evidenced by elevated blood urea nitrogen (2.50 ± 0.46 vs. 11.61 ± 1.61 mmol/L, *p* < 0.001), serum creatinine (43.01 ± 5.81 vs. 107.62 ± 9.90 μmol/L, *p* < 0.001), and urine albumin‐to‐creatinine ratio (18.53 ± 0.92 vs. 269.97 ± 24.59 mg/g, *p* < 0.001). Fer‐1 treatment significantly ameliorated these parameters (*p* < 0.05) and reduced histopathological damage. DN rats exhibited significantly reduced DUSP1 expression and increased ferroptosis‐associated alterations, including elevated ACSL4 expression, enhanced lipid peroxidation, and impaired antioxidant capacity, all of which were partially reversed by Fer‐1 treatment (*p* < 0.001).

**Conclusions:**

Our findings indicate that inhibition of ferroptosis attenuates renal injury in DN and is accompanied by altered DUSP1 expression. These results suggest a potential association between ferroptosis regulation and DUSP1 in DN, providing new insight into ferroptosis‐related mechanisms involved in disease progression.

## Graphical abstract

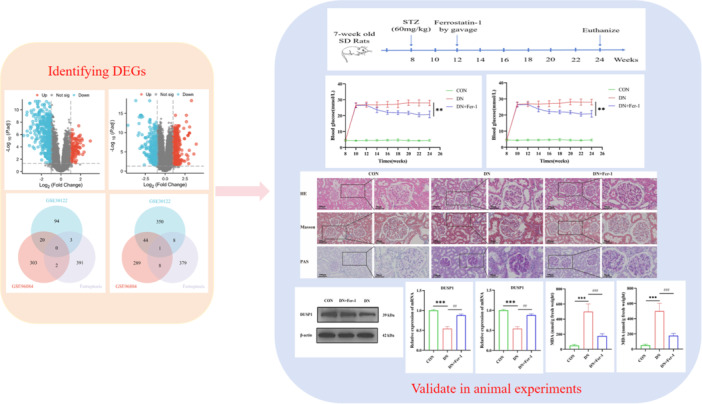

**Abbreviations**
BUN: blood urea nitrogen
DN: diabetes nephropathy
DUSP1: dual specificity phosphatase 1
FDEGs: ferroptosis related differentially expressed genes
GEO: gene expression omnibus
GSH: reduced glutathione
MDA: malondialdehyde
Scr: serum creatinine
SD: Sprague Dawley
SOD: superoxide dismutase
STZ: streptozotocin
UACR: urine albumin‐to‐creatinine ratio

## 1 Introduction

Diabetic nephropathy (DN), a prevalent microvascular complication of diabetes mellitus, has emerged as the leading cause of chronic kidney disease in China, accounting for approximately 40% of end‐stage renal disease cases [1, 2]. Beyond a metabolic disorder, DN is increasingly recognized as a disease process in which inflammatory responses contribute to disease progression within the renal microenvironment. Despite advances in glycemic control and antihypertensive therapies, nearly 40% of diabetic patients still progress to end‐stage renal disease, underscoring an urgent need to identify novel immuno‐inflammatory mechanisms and therapeutic targets [3].

The pathological hallmarks of DN—including glomerulosclerosis, tubular epithelial injury, interstitial fibrosis, and progressive renal dysfunction—are tightly associated with persistent inflammatory activation [4]. Accumulating evidence indicates that chronic hyperglycemia induces oxidative stress, which not only causes direct cellular damage but also acts as a potent trigger of inflammatory signaling [5, 6]. Excessive production of reactive oxygen species (ROS) promotes lipid peroxidation, protein modification, and DNA damage, while simultaneously activating redox‐sensitive transcription factors such as nuclear factor kappa‐B (NF‐κB), leading to sustained expression of pro‐inflammatory cytokines including tumor necrosis factor‐alpha and interleukin‐6 (IL‐6) [7, 8]. This reciprocal amplification between oxidative stress and inflammation establishes a self‐perpetuating cycle that accelerates renal injury and immune imbalance. Notably, the limited efficacy of conventional antioxidant therapies in clinical trials suggests that alternative, inflammation‐linked cell death mechanisms may play a critical role in DN progression [9].

Ferroptosis, a recently identified iron‐dependent form of regulated cell death, has emerged as a key intersection between oxidative stress and inflammation [10, 11]. Characterized by mitochondrial shrinkage, increased membrane density, and loss of cristae, ferroptosis is initiated by iron‐catalyzed lipid peroxidation and impaired antioxidant defense [12]; two central mechanisms underlie this process: excessive peroxidation of polyunsaturated fatty acids mediated by iron‐dependent enzymes, and inactivation of glutathione peroxidase 4 (GPX4), the principal enzyme responsible for detoxifying lipid hydroperoxides [13]. Importantly, ferroptotic signaling has been shown to actively propagate inflammatory responses through the release of damage‐associated molecular patterns and modulation of immune cell activation, positioning ferroptosis as both a consequence and a driver of chronic inflammation.

Renal iron homeostasis is particularly vulnerable to dysregulation in diabetes. Increased expression of transferrin receptor 1 promotes intracellular iron accumulation, fueling Fenton reaction‐mediated ROS generation and lipid peroxidation in renal cells [14]. Preclinical studies consistently report GPX4 downregulation and elevated ferroptosis markers in diabetic kidneys [15], while clinical evidence demonstrates iron overload and heightened oxidative stress in patients with type 2 diabetes [16]. These findings collectively suggest that ferroptosis represents a critical immuno‐inflammatory effector mechanism in DN.

Dual specificity phosphatase 1 (DUSP1), also known as MAP kinase phosphatase‐1, is an inducible nuclear phosphatase that negatively regulates stress‐activated mitogen‐activated protein kinase (MAPK) pathways, including p38, JNK, and ERK [17]. In the kidney, DUSP1 is expressed in multiple renal cell types, such as tubular epithelial cells, mesangial cells, and podocytes, where it plays a critical role in modulating inflammatory and stress responses [18]. Emerging evidence indicates that DUSP1 functions as an endogenous protective regulator during renal injury. Its expression is rapidly induced by oxidative stress and inflammatory stimuli, acting as a negative feedback mechanism to restrain excessive MAPK‐driven cytokine production [19]. Loss or downregulation of DUSP1 has been associated with aggravated renal inflammation, enhanced tubular injury, and increased susceptibility to fibrosis in experimental models of kidney disease [20, 21].

Despite these observations, the role of DUSP1 in DN, particularly in the context of ferroptosis‐associated oxidative injury and chronic inflammation, remains poorly defined. Whether DUSP1 participates in modulating ferroptosis‐related renal damage or reflects a compensatory response to ferroptotic stress in the diabetic kidney has not been systematically explored.

**Figure 1:**
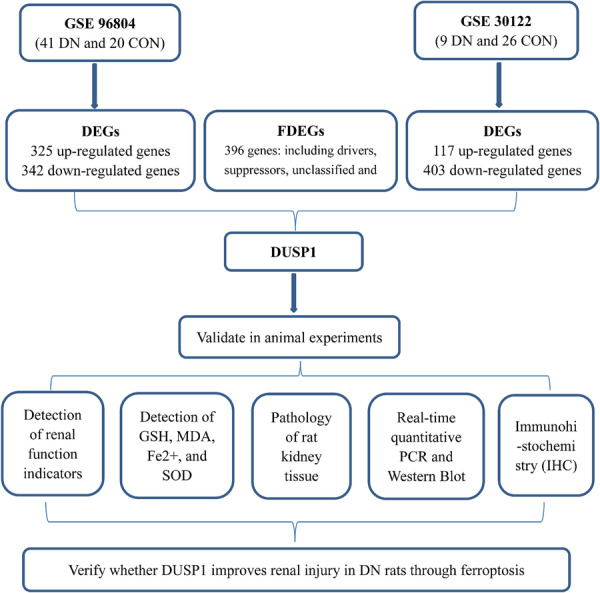
Schematic workflow of the study. Two diabetic nephropathy (DN) microarray datasets (GSE96804 and GSE30122) were analyzed to identify differentially expressed genes (DEGs). Ferroptosis‐related differentially expressed genes (FDEGs) were obtained by intersecting DEGs with genes curated in the FerrDb database, from which DUSP1 was identified as a candidate gene associated with DN. Subsequent experimental validation was performed in a streptozotocin‐induced DN rat model, including assessment of renal function parameters, ferroptosis‐related markers (GSH, MDA, Fe²⁺, and SOD), renal histopathology, and DUSP1 expression by quantitative PCR, Western blotting, and immunohistochemistry. This workflow illustrates the integrative bioinformatics and experimental approach used to explore the association between ferroptosis and DUSP1 in DN.

Given the mechanistic convergence of immune activation, chronic inflammation, and ferroptotic cell death in DN, we hypothesized that targeting ferroptosis‐related regulatory pathways may offer a novel strategy to attenuate renal injury. In this study, we adopted a translational approach integrating bioinformatics analysis of DN‐associated ferroptosis genes from GEO datasets with experimental validation in a streptozotocin‐induced DN rat model. We specifically focused on DUSP1, a key modulator of inflammatory and stress‐responsive signaling, to elucidate its potential role in linking ferroptosis regulation with renal immune homeostasis. The overall study design is illustrated in Figure 1.

## 2 Materials and Methods

### 2.1 Data Acquisition and Preprocessing

We obtained two microarray datasets (GSE30122 and GSE96804) related to DN from the gene expression omnibus (GEO) database (https://www.ncbi.nlm.nih.gov/geo/) [22], which included 9 DN and 26 control samples in GSE30122, and 41 DN and 20 control samples in GSE96804. To ensure data comparability, we employed the sva package (v3.52.4) [23] in R for batch effect correction and used the k‐nearest neighbor algorithm [24] for missing value imputation, while averaging expression values from multiple probes targeting the same gene symbol to obtain representative expression values. Differential expression analysis was performed separately for each dataset using the limma package with a significance threshold of |log₂ fold change | > 1 and Benjamini‐Hochberg adjusted *p*‐value < 0.05, with results visualized through volcano plots generated by ggplot2.

### 2.2 Ferroptosis‐Related Differential Expressed Genes (FDEGs)

We systematically retrieved ferroptosis‐related genes (including drivers, suppressors, markers, and unclassified genes) from the FerrDb database (https://www.zhounan.org/ferrdb/current) [25], a manually curated knowledgebase for ferroptosis research, and subsequently employed a Venn diagram analysis (http://www.ehbio.com/test/venn/#/) to identify the overlapping differentially expressed genes (DEGs) and FDEGs that exhibited consistent upregulation or downregulation patterns across both datasets.

### 2.3 Experimental Reagents

All chemical reagents and experimental materials were obtained from commercial sources: Ferrostatin‐1 (Fer‐1) (GC10380, Glpbio, California, USA); β‐actin primary antibodies and HRP‐conjugated goat anti‐rabbit/anti‐mouse IgG secondary antibodies (66009‐1‐Ig and RGAR001, respectively; Proteintech, Wuhan, China); DUSP1 antibodies (AF5286, Affinity, Shanghai, China); ACSL4 antibodies (ET7111‐43, Huabio, Hangzhou, China); streptozotocin (Sigma‐Aldrich, St. Louis, MO, USA); histological staining kits for Hematoxylin and Eosin (HE), Masson's Trichrome (Masson), PAS Stain, and Lillie's ferrous iron staining (G1120, G1340, G1281, and G3320, respectively; Solarbio, Beijing, China); and biochemical assay kits for MDA, reduced glutathione (GSH), superoxide dismutase (SOD), and Fe²⁺ quantification (G0109W48, G0206W48, G0101W48, and G1217W, respectively; Suzhou Grace Biotechnology Co. Ltd., China).

### 2.4 Animal Model Establishment

Male *Sprague‐Dawley* rats (8–10 weeks old, 180–220 g) were obtained from Jiangsu Jicui Yaokang Biotechnology Co. Ltd. (License No.: SCXK(Su)2023‐0009) and acclimatized for 1 week under specific pathogen‐free conditions (21°C–23°C, 50%–60% humidity, 12‐h light/dark cycle). Following overnight fasting, diabetes was induced in 30 rats via intraperitoneal injection of streptozotocin (STZ, 60 mg/kg in citrate buffer, pH 4.5), while 15 control rats received vehicle only. After 72 h, diabetes induction was confirmed by three consecutive measurements of tail vein blood glucose > 16.7 mmol/L. Successful DN modeling (93.33% success rate, *n* = 28/30) was further verified after 12 weeks by urine albumin‐to‐creatinine ratio (UACR) ≥ 30 mg/g. The DN rats were then randomly allocated to DN (*n* = 14) and DN + Fer‐1 (*n* = 14) groups, with the latter receiving daily oral gavage of Fer‐1 (2.5 μmol/kg) for 12 weeks, while controls (*n* = 15) and DN groups received deionized water. Throughout the study, conducted in compliance with international animal care standards (Approval No.: CDYFY‐IACUC‐202401QR003, The First Affiliated Hospital of Nanchang University), all rats had ad libitum access to food/water, with weekly monitoring of body weight, blood glucose, and metabolic parameters, culminating in tissue collection from control (*n* = 15), DN (*n* = 14), and DN+Fer‐1 (*n* = 13) groups for analysis.

### 2.5 Specimen Collection and Processing

Daily monitoring of all rats included comprehensive assessments of general health status (including mental alertness, physical activity, fur condition, and survival), with weekly measurements of body weight and blood glucose every Saturday, accompanied by detailed recording of 24‐h water intake and urine output using metabolic cages. All animals were anesthetized by intraperitoneal injection of sodium pentobarbital (30–50 mg/kg) before invasive procedures. At the end of the experiment, animals were humanely euthanized by an overdose of sodium pentobarbital (100–150 mg/kg, intraperitoneally) in accordance with institutional guidelines. Death was confirmed by complete cessation of heartbeat and respiration for at least 5 min, as well as the absence of a corneal reflex. Blood samples were collected by cardiac puncture, centrifuged at 4°C and 1000 × g for 20 min, and the supernatant was stored at −80°C for further analysis. Through aseptic laparotomy, bilateral kidneys were carefully excised, rinsed with ice‐cold physiological saline (4°C), and divided for processing: one portion was fixed in 4% paraformaldehyde for paraffin embedding, sectioning (5 μm thickness), and histological staining (HE, Masson, PAS), while the remaining tissue was flash‐frozen in liquid nitrogen and stored at −80°C for molecular analyses.

### 2.6 Assessment of Renal Function Parameters

Upon completion of the experimental protocol, renal function was evaluated by collecting terminal blood samples via cardiac puncture and 24‐h urine specimens using metabolic cages, with subsequent biochemical analysis performed at the First Affiliated Hospital of Nanchang University using a fully automated clinical chemistry analyzer to quantify key renal function markers including serum creatinine (Scr), blood urea nitrogen (BUN), and UACR.

### 2.7 Quantification of Oxidative Stress Markers in Renal Tissue

Renal tissue samples stored at −80°C were homogenized in ice‐cold 0.9% physiological saline (1:9 w/v) using a glass homogenizer maintained on ice, followed by centrifugation at 3000 rpm for 15 min at 4°C to obtain 10% tissue homogenate supernatant. For analyte extraction, 100 μL of homogenate was mixed with 100 μL precipitant and centrifuged at 3500 rpm for 10 min at 4°C. According to the instructions of the reagent kit, add samples in sequence for testing: set up three parallel wells for each group of samples, add samples in sequence, and mix well. OD values were measured at the appropriate wavelengths according to the manufacturer's instructions for each assay.

### 2.8 Histopathological Examination of Renal Tissue

Fresh kidney tissues were immediately fixed in 4% paraformaldehyde for 48 h, followed by sequential dehydration through graded ethanol solutions (70%, 80%, 95%, and 100%), paraffin embedding using standard protocols, and sectioning at 5 μm thickness with a rotary microtome. The sections were mounted on glass slides, dried at 65°C for 2 h, and subsequently stained with HE, Masson's trichrome, PAS, and Perl's Prussian blue (Lillie's method) according to manufacturer's instructions, with all stained sections being stored under controlled conditions (25°C, 40% humidity) until microscopic evaluation.

### 2.9 Quantitative Real‐Time Polymerase Chain Reaction (PCR) Analysis

Total RNA was isolated from renal tissues using TRIzol reagent (Invitrogen, Thermo Fisher Scientific, USA), and RNA purity and concentration were verified spectrophotometrically (A260/A280 ratio > 1.8). Complementary DNA (cDNA) was synthesized by reverse transcription using a commercial kit (TransScript Uni All‐in‐One First‐Strand cDNA Synthesis SuperMix, TransGen Biotech, China). Quantitative real‐time PCR was performed to assess the mRNA expression levels of ferroptosis‐ and inflammation‐related genes, including DUSP1 and ACSL4, using a CFX96 Real‐Time PCR Detection System (Bio‐Rad, USA) with TransStart Top Green qPCR SuperMix (TransGen Biotech, China). All reactions were carried out in triplicate under the following cycling conditions: 94°C for 30 s, followed by 40 cycles of 94°C for 5 s and 60°C for 30 s. Primer sequences are listed in Supporting Information: Table S1. Relative gene expression levels were calculated using the 2^(−ΔΔCt) method and normalized to GAPDH as the endogenous control. Statistical significance was determined using Student's *t*‐test, with *p* < 0.05 considered statistically significant.

### 2.10 Western Blot Analysis

Total protein extracts were resolved on 10% or 12.5% SDS‐polyacrylamide gels (SDS‐PAGE) and subsequently transferred onto polyvinylidene difluoride membranes using standard electroblotting procedures. Following blocking with 5% nonfat milk in TBST (Tris‐buffered saline containing 0.1% Tween‐20) for 1 h at room temperature, membranes were incubated overnight at 4°C with primary antibodies against DUSP1 (1:1000, AF5286, Affinity), ACSL4 (1:1000, ET7111‐43, Huabio), and β‐actin (1:5000, Proteintech) as loading control. After three 10‐min washes with TBST, membranes were probed with appropriate horseradish peroxidase (HRP)‐conjugated secondary antibodies (1:5000, Proteintech) for 1 h at room temperature. Protein bands were visualized using enhanced chemiluminescence (ECL‐plus, Thermo Fisher Scientific, USA) and quantified by densitometry with ImageJ software (NIH, Bethesda, MD), with all results normalized to β‐actin expression levels.

### 2.11 Immunohistochemical Analysis of DUSP1 Expression

Renal tissues from control (*n* = 15), DN (*n* = 14), and DN+Fer‐1 (*n* = 14) groups were fixed in 4% paraformaldehyde, dehydrated through graded ethanol series, embedded in paraffin, and sectioned at 5 μm thickness for immunohistochemical analysis. Following deparaffinization and rehydration, sections underwent antigen retrieval in citrate buffer (pH 6.0), endogenous peroxidase blocking with 3% H_2_O_2_, and nonspecific binding blocking with 5% goat serum (20 min, room temperature). Tissues were then incubated with anti‐DUSP1 primary antibody (1:200 dilution, 4°C, 12 h), washed with PBS, and probed with biotinylated secondary antibody (40 min, room temperature). After DAB chromogenic development (5–10 min) and hematoxylin counterstaining, sections were dehydrated, cleared in xylene, and mounted with neutral gum. Quantitative analysis was performed using ImageJ software by measuring average optical density in at least three randomly selected glomerular and tubular regions per section, with final values representing the mean of all measurements per experimental group.

### 2.12 Statistical Analysis

All statistical analyses were performed using GraphPad Prism software (version 8.3; GraphPad Software, San Diego, CA), with quantitative data from qRT‐PCR and immunohistochemistry (IHC) expressed as mean ± standard deviation (SD) after verifying normal distribution using the Kolmogorov–Smirnov test, where normally distributed data were analyzed by Student's *t*‐test and nonparametric data by Mann–Whitney *U*‐test, with a threshold of *p* < 0.05 considered statistically significant for all comparisons between experimental groups.

## 3 Results

### 3.1 Identification of DEGs

**Figure 2:**
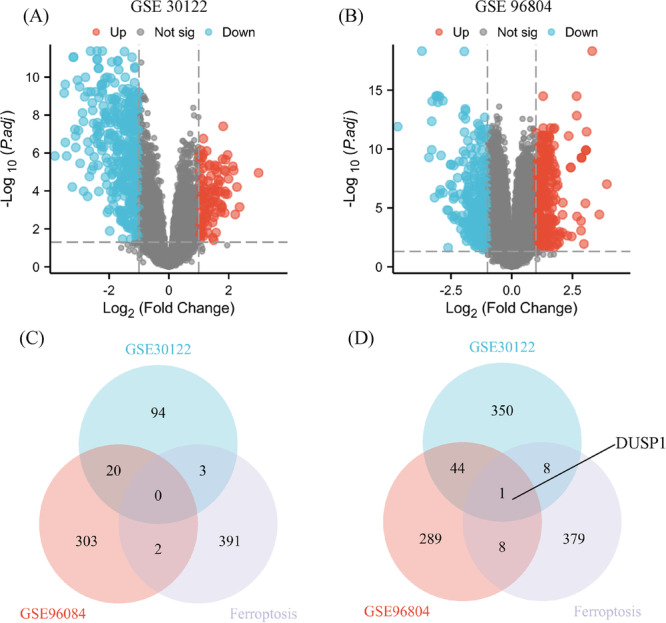
Identification of ferroptosis‐related differentially expressed genes (DEGs) in diabetic nephropathy. (A) Volcano plot of DEGs in the GSE30122 dataset. Genes with |log₂ fold change | > 1 and adjusted *p* < 0.05 were considered significantly differentially expressed (red, upregulated; blue, downregulated). (B) Volcano plot of DEGs in the GSE96804 dataset using the same screening criteria as in panel (A). (C) Venn diagram showing the overlap between co‐upregulated DEGs identified from GSE30122 and GSE96804 and ferroptosis‐related genes curated in the FerrDb database. (D) Venn diagram showing the overlap between co‐downregulated DEGs from GSE30122 and GSE96804 and ferroptosis‐related genes.

Microarray datasets GSE30122 (9 DN vs. 26 controls) and GSE96804 (41 DN vs. 20 controls) were retrieved from the GEO database, with differential expression analysis (adjusted *p* < 0.05, |log₂FC | > 1.0) identifying 520 DEGs (117 up/403 down) in GSE30122 and 667 DEGs (325 up/342 down) in GSE96804 (Figure 2A,B). Comparative analysis revealed DUSP1 as the sole overlapping gene between the ferroptosis database and consistently downregulated genes across both datasets (Figure 2C,D), establishing its potential role in ferroptosis‐mediated DN pathogenesis.

### 3.2 Results of Animal Experiments

#### 3.2.1 Behavioral and Metabolic Observations

Control rats displayed normal behavioral characteristics including alertness, active movement, glossy fur, and stable metabolic parameters (food/water intake and urine output). In contrast, DN model rats exhibited marked lethargy, reduced responsiveness to stimuli, dull fur coat, and significantly increased food/water consumption (polyphagia/polydipsia) and urine production (polyuria). Fer‐1‐treated rats showed intermediate phenotypes, with partial restoration of normal activity levels, improved fur condition, and moderated food/water intake and urine output compared to untreated DN rats.

#### 3.2.2 Metabolic Parameters and Body Weight Changes

**Figure 3:**
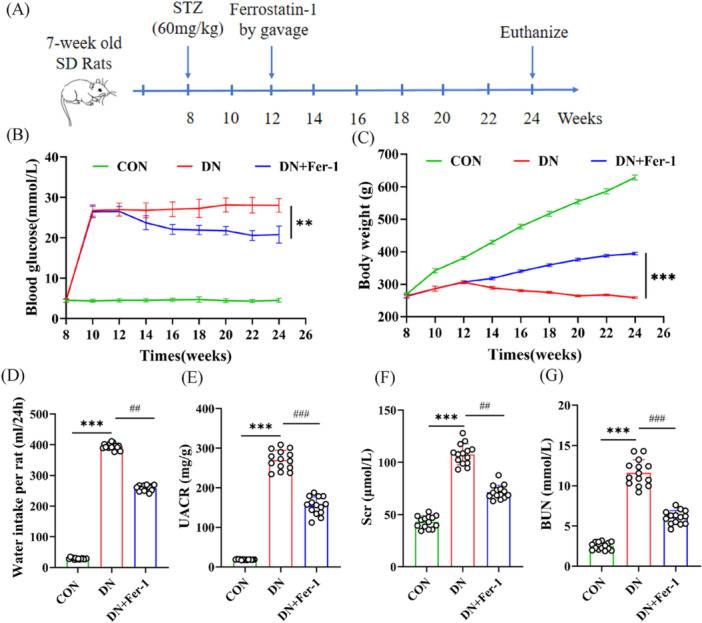
Effects of ferrostatin‐1 on metabolic parameters and renal function in diabetic rats. (A) Experimental timeline illustrating the induction of diabetic nephropathy by streptozotocin (STZ, 60 mg/kg), the administration of ferrostatin‐1 (Fer‐1), and the schedule for sample collection and euthanasia. (B) Blood glucose levels over time in control (CON), diabetic nephropathy (DN), and DN + Fer‐1 groups. (C) Changes in body weight during the experimental period. (D) Comparison of 24‐h water intake among the different groups. (E–G) Urine albumin‐to‐creatinine ratio (UACR), serum creatinine (Scr), and blood urea nitrogen (BUN) across the indicated groups. Data are presented as individual data points with bars indicating mean ± SD. Statistical significance was determined as follows: compared with the control group, **p < 0.01, ***p < 0.001; compared with the DN group, ^##^ *p* < 0.01, ^###^ *p* < 0.001.

Figure 3A presents a schematic timeline outlining the entire experimental protocol for the animal study. Longitudinal monitoring demonstrated that DN model rats developed characteristic metabolic derangements, including: (1) severe hyperglycemia (28.00 ± 1.69 mmol/L vs. 4.53 ± 0.53 mmol/L in controls, *p* < 0.001); (2) significant weight reduction (259.28 ± 2.89 g vs. 628.43 ± 7.53 g in controls, *p* < 0.001); and (3) marked polydipsia (water intake: 394.32 ± 9.92 mL/day vs. 28.84 ± 2.45 mL/day in controls, *p* < 0.001); (Figure 3B–D). Fer‐1 intervention produced significant therapeutic effects, attenuating hyperglycemia by 25.8% (20.78 ± 2.12 mmol/L, *p* < 0.001), restoring 52.1% of lost body weight (394.50 ± 4.01 g, *p* < 0.001), and reducing water intake by 34.5% (258.41 ± 8.89 mL/day, *p* < 0.001) compared to untreated DN rats. These findings collectively demonstrate Fer‐1's capacity to ameliorate core diabetic manifestations through ferroptosis pathway modulation.

#### 3.2.3 Renal Function Assessment

Quantitative analysis of renal function biomarkers revealed significant impairment in DN rats, demonstrating elevated urinary albumin‐to‐creatinine ratio (UACR: 18.53 ± 0.92 vs. 269.97 ± 24.59 mg/g, *p* < 0.001), increased (BUN: 2.50 ± 0.46 vs. 11.61 ± 1.61 mmol/L, *p* < 0.001), and higher Scr levels (Scr: 43.01 ± 5.81 vs. 107.62 ± 9.90 μmol/L, *p* < 0.001) compared to controls (Figure 3E–G). Fer‐1 treatment significantly attenuated these pathological changes, reducing UACR by 42.8% (154.45 ± 22.38 mg/g, *p* < 0.001), BUN by 47.7% (6.07 ± 0.84 mmol/L, *p* < 0.001), and Scr by 33.5% (71.59 ± 6.55 μmol/L, *p* < 0.001) relative to untreated DN rats, indicating substantial renoprotective effects through ferroptosis inhibition.

#### 3.2.4 Histopathological Characterization of Renal Injury

**Figure 4:**
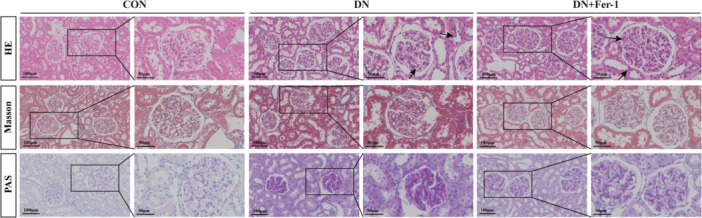
Histopathological changes in renal tissues of rats from different groups. Representative kidney sections were stained sequentially with hematoxylin and eosin (H&E), Masson's trichrome, and periodic acid–Schiff (PAS) to evaluate renal pathological alterations. Low‐magnification images (× 20) provide an overview of renal morphology, while higher‐magnification images (× 50) highlight representative pathological features. In H&E‐stained sections, arrows indicate areas of glomerular and tubular injury, inflammatory cell infiltration, and structural disorganization. Masson's trichrome staining was used to visualize collagen deposition and interstitial fibrosis, whereas PAS staining was applied to assess mesangial matrix expansion and glomerular basement membrane thickening. Scale bars are shown in each panel.

Comprehensive histopathological analysis using hematoxylin‐eosin (HE), Masson's trichrome, and periodic acid‐Schiff (PAS) staining demonstrated distinct morphological alterations across experimental groups (Figure 4). Control rats exhibited normal renal architecture, featuring intact tubular epithelium, well‐defined glomerular capillaries, and minimal collagen deposition. In contrast, DN rats displayed hallmark pathological changes including glomerular hypertrophy, tubular atrophy, extensive interstitial fibrosis, and pronounced leukocyte infiltration. Fer‐1 treatment significantly ameliorated these abnormalities, restoring glomerular morphology, decreasing fibrosis, and reducing inflammatory infiltration, indicating substantial tissue protection through ferroptosis modulation.

#### 3.2.5 Renal Iron Homeostasis Assessment

**Figure 5:**
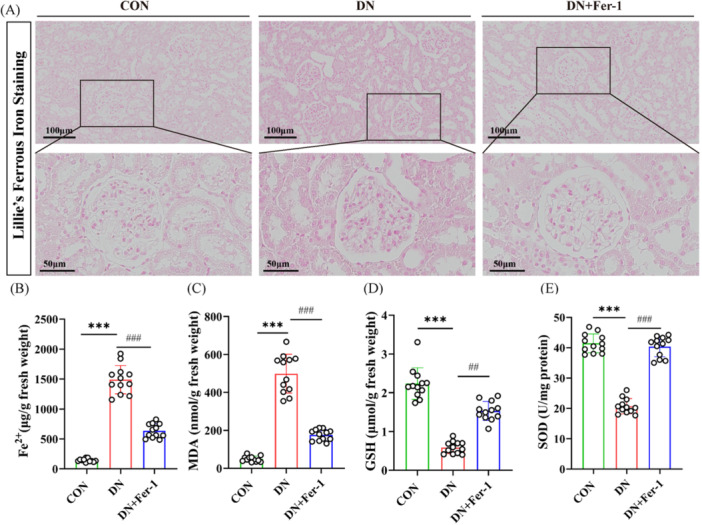
Changes in ferrous iron content and oxidative stress markers in renal tissues of rats across different groups. (A, B) Representative kidney sections stained with Lillie's ferrous iron staining showing Fe²⁺ deposition. Images were acquired at low magnification (× 20) and high magnification (× 60). Minimal iron staining was observed in the control group, whereas increased Fe²⁺ deposition was detected in diabetic nephropathy (DN) rats. Fer‐1 treatment was associated with a reduction in renal iron accumulation in DN rats. Scale bars are shown in each panel. (C–E) Malondialdehyde (MDA) levels, glutathione (GSH) content, and superoxide dismutase (SOD) activity in renal tissues from different experimental groups. Data are presented as individual data points with bars indicating mean ± SD. Statistical significance was defined as follows: compared with the control group, ***p < 0.001; compared with the DN group, ^##^ *p* < 0.01, ^###^ *p* < 0.001.

Quantitative analysis of renal iron content revealed significant dysregulation in DN progression. Lillie's staining (Figure 5A) demonstrated minimal Fe²⁺ deposition in control rats, whereas DN rats exhibited marked iron accumulation, particularly in proximal tubules. Fer‐1 treatment significantly attenuated this iron overload. Biochemical assays corroborated these findings, showing a 10.27‐fold increase in renal Fe²⁺ content in DN rats (1490.39 ± 236.29 vs. 145.02 ± 26.41 μg/g fresh weight in controls, *p* < 0.001), which was reduced by 57.3% following Fer‐1 intervention (635.28 ± 117.88 μg/g fresh weight, *p* < 0.001) (Figure 5B). These results establish that: (1) iron dyshomeostasis is a hallmark of DN pathology; and (2) Fer‐1 effectively restores renal iron metabolism through ferroptosis inhibition.

#### 3.2.6 Oxidative Stress Markers in Renal Tissues

Quantitative analysis of oxidative stress biomarkers revealed significant alterations in DN rats, demonstrating a 10.13‐fold increase in malondialdehyde (MDA) levels (498.60 ± 102.29 vs. 49.19 ± 14.75 nmol/g fresh weight in controls, *p* < 0.001), along with markedly depleted antioxidant defenses as evidenced by 49.9% reduction in SOD activity (20.72 ± 2.51 vs. 41.43 ± 3.11 U/mg protein, *p* < 0.001) and 73.6% decrease in reduced GSH content (0.59 ± 0.16 vs. 2.24 ± 0.41 μmol/g fresh weight, *p* < 0.001) compared to controls (Figure 5C–E); Fer‐1 treatment effectively mitigated these oxidative perturbations, reducing MDA accumulation by 64.7% (175.96 ± 29.31 nmol/g fresh weight, *p* < 0.001 vs. DN group), while restoring SOD activity (40.35 ± 3.35 U/mg protein, 97% of control levels) and GSH content (1.53 ± 0.25 μmol/g fresh weight, 68% of control levels) to near‐normal ranges (all *p* < 0.01), indicating potent antioxidative effects through ferroptosis inhibition.

#### 3.2.7 Quantitative Profiling of Renal DUSP1 and ACSL4 Expression

**Figure 6:**
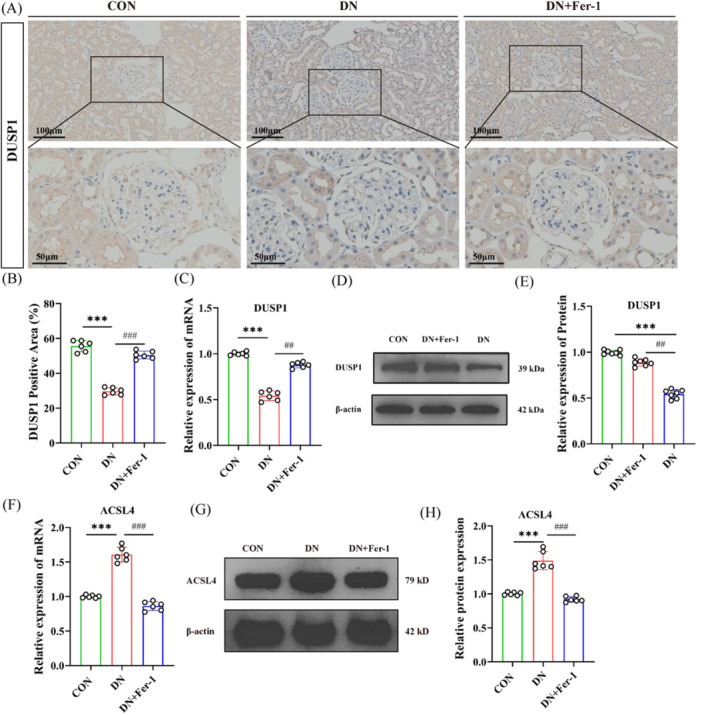
Changes in DUSP1 and ACSL4 expression in renal tissues of rats across different groups. (A) Representative immunohistochemical (IHC) staining of DUSP1 in renal tissues from different experimental groups. Images were acquired at low magnification (× 20) and high magnification (× 60). (B) Quantitative analysis of DUSP1‐positive area in renal tissues based on IHC staining shown in panel (A). (C, F) Relative mRNA expression levels of DUSP1 and ACSL4 in renal tissues determined by quantitative real‐time PCR (qPCR). (D, G) Representative Western blot (WB) images showing protein expression levels of DUSP1 and ACSL4 in renal tissues. (E, H) Densitometric quantification of DUSP1 and ACSL4 protein expression based on the Western blot results shown in panel (D). Data are presented as individual data points with bars indicating mean ± SD. Statistical significance was defined as follows: compared with the control group, *p < 0.05, **p < 0.01, ***p < 0.001; compared with the DN group, ^#^ *p* < 0.05, ^##^ *p* < 0.01, ^###^ *p* < 0.001.

Comprehensive molecular analyses, including IHC, quantitative real‐time PCR (qPCR), and Western blotting, were performed to characterize the expression patterns of DUSP1 and the ferroptosis‐related enzyme ACSL4 in renal tissues across different experimental groups (Figure 6). IHC staining demonstrated a marked reduction in DUSP1 protein expression in the kidneys of DN rats compared with control animals (Figure 6A,B). Quantitative analysis revealed a significant decrease in the DUSP1‐positive area in DN rats, which was partially restored following Fer‐1 treatment. Consistently, qPCR analysis showed that renal DUSP1 mRNA levels were significantly downregulated in the DN group and were markedly upregulated after Fer‐1 intervention (Figure 6C). Western blot analysis further confirmed these findings at the protein level, demonstrating reduced DUSP1 expression in DN rats and a pronounced recovery upon Fer‐1 treatment (Figure 6D–E). In parallel, the expression of ACSL4, a key enzyme involved in ferroptosis‐associated lipid peroxidation, was examined. qPCR analysis revealed a significant increase in ACSL4 mRNA expression in renal tissues from DN rats compared with controls, whereas Fer‐1 treatment markedly attenuated this upregulation (Figure 6F). Western blot analysis showed a corresponding increase in ACSL4 protein levels in DN rats, which was significantly reduced following Fer‐1 administration, as confirmed by densitometric quantification (Figure 6G,H).

Collectively, these results demonstrate that DN is characterized by coordinated downregulation of DUSP1 and upregulation of ACSL4 in renal tissues, and that Fer‐1 treatment simultaneously restores DUSP1 expression while suppressing ACSL4 induction, consistent with inhibition of ferroptosis‐related molecular alterations.

## 4 Discussion

DN remains the leading cause of end‐stage renal disease worldwide and is characterized by complex pathophysiology and steadily increasing incidence rates [26]. Despite extensive preclinical investigations, the translational value of animal studies is often limited by variability in experimental models, including differences in animal age, baseline renal function, and treatment regimens [27]. Moreover, current clinical strategies primarily targeting glycemic control, blood pressure regulation, and renin–angiotensin system blockade have only modest effects on halting DN progression, underscoring the urgent need to identify novel pathogenic mechanisms and therapeutic targets.

Ferroptosis, an iron‐dependent form of regulated cell death distinct from apoptosis and necrosis, is characterized by iron overload, excessive lipid peroxidation, and depletion of antioxidant capacity [11]. Increasing evidence implicates ferroptosis in the pathogenesis of diabetes and its complications, including DN, through disruption of renal iron homeostasis and redox balance [28]. In the present study, DN rats exhibited pronounced iron accumulation, enhanced lipid peroxidation, and impaired antioxidant defenses, consistent with ferroptotic injury. Importantly, pharmacological inhibition of ferroptosis with Fer‐1 markedly attenuated these alterations and improved renal histopathology and functional parameters, supporting a contributory role of ferroptosis in DN progression.

To further substantiate ferroptosis activation, we examined ACSL4, a key executor of ferroptosis that facilitates polyunsaturated fatty acid incorporation into membrane phospholipids and promotes lipid peroxidation [29, 30]. Our results demonstrated that ACSL4 expression was markedly upregulated in the kidneys of DN rats and significantly suppressed following Fer‐1 treatment. These findings provide additional molecular evidence linking ferroptotic lipid peroxidation to renal injury in DN and further substantiate the involvement of ferroptosis‐related pathways in this disease context.

A key observation of this study is that modulation of ferroptosis was accompanied by parallel changes in DUSP1 expression. Renal DUSP1 levels were reduced in DN and partially restored following Fer‐1 treatment. DUSP1 is a well‐recognized stress‐responsive phosphatase and a negative regulator of MAPK signaling, with documented roles in limiting renal fibrosis, inflammatory activation, and macrophage infiltration [31, 32, 33]. While previous studies have primarily focused on the anti‐inflammatory and anti‐fibrotic functions of DUSP1, our findings extend these observations by demonstrating an association between ferroptotic injury and altered DUSP1 expression in the diabetic kidney. Notably, as DUSP1 was not directly manipulated in the present study, the observed changes are more likely to reflect a secondary adaptive or stress‐related response to ferroptotic injury rather than a primary regulatory role. Therefore, causality between DUSP1 and ferroptosis cannot be inferred from the current data.

In addition to oxidative damage, chronic inflammation is a central feature of DN pathogenesis [34]. Our results indicate that inhibition of ferroptosis is accompanied by attenuation of inflammatory responses, as evidenced by reduced inflammatory markers and improved renal histological features. This observation is consistent with emerging concepts that ferroptotic cell injury may amplify inflammatory processes within the renal microenvironment. However, because upstream inflammatory signaling pathways such as MAPK or NF‐κB were not directly interrogated in this study, mechanistic interpretations regarding these pathways were intentionally restrained.

Current therapeutic strategies for DN, including sodium–glucose cotransporter 2 (SGLT2) inhibitors and renin–angiotensin system blockers, primarily address metabolic and hemodynamic disturbances but do not directly target regulated cell death mechanisms [35]. In this context, our findings highlight ferroptosis inhibition as a potential complementary therapeutic strategy. The renoprotective effects of Fer‐1 observed in this study are consistent with prior reports demonstrating protective roles of ferroptosis inhibition in renal injury models [36]. Nevertheless, the short half‐life of Fer‐1 limits its translational applicability, and future studies should explore alternative ferroptosis inhibitors or iron‐modulating strategies with improved pharmacokinetic properties. In parallel, the observed modulation of DUSP1 expression suggests that stress‐responsive phosphatases may represent additional points of intervention, although their precise functional roles require further clarification.

This study has several limitations that should be acknowledged. First, the experiments were conducted exclusively in a rat model of DN. Although this model is widely used and recapitulates key pathological features of the disease, it may not fully reflect the complexity and heterogeneity of human diabetic kidney disease. Therefore, caution is required when extrapolating these findings to clinical settings. Second, this study primarily focused on structural, molecular, and biochemical indicators of renal injury, while comprehensive assessments of functional outcomes at the cellular level were not extensively performed. Future studies incorporating additional functional assays may further strengthen the interpretation of the findings. Third, the duration of the study represents an inherent limitation. The evaluation period was limited to 12 weeks, which mainly reflects the early to middle stages of DN. Longer‐term studies are needed to determine whether DUSP1 provides sustained renoprotection during advanced disease stages, particularly in the context of progressive fibrosis and renal functional decline.

## 5 Conclusion

In summary, this study demonstrates that pharmacological inhibition of ferroptosis attenuates renal injury in DN, accompanied by reduced oxidative damage and modulation of inflammatory responses. Notably, changes in DUSP1 expression were observed in parallel with ferroptosis inhibition, suggesting a potential association between ferroptotic signaling and DUSP1 in the diabetic kidney. Although the precise mechanistic relationship remains to be elucidated, these findings provide a basis for further investigation into ferroptosis‐related pathways in DN and highlight the need for future studies employing direct genetic or pharmacological manipulation of DUSP1 to clarify its functional role.

## Author Contributions

Jixiong Xu conceived and designed the study. Jiarong Liu and Junping Zhang extracted the data and performed the analyses. Yun Zou and Jiarong Liu conducted the experiments. Jiarong Liu and Wen Chen drafted the manuscript. Jixiong Xu and Jiarong Liu revised the manuscript.

## Conflicts of Interest

The authors declare no conflicts of interest.

## Supporting information


**Supplementary Table S1:** Primer sequence of quantitative real‐time PCR.

## Data Availability

The datasets analyzed in our study were downloaded from the GEO database, and the datasets used during the current study are available from the corresponding author on reasonable request.
