# Supplementary material for: DUSP1 Attenuates Renal Injury in Diabetic Nephropathy by Modulating Ferroptosis: Evidence From Animal Experiments
Source: Immun Inflamm Dis. 2026 Feb 4;14(2):e70340. doi: 10.1002/iid3.70340 (PMC12872968; doi:10.1002/iid3.70340)
Supplement: Supplementary file 1 — Supplementary Table S1: Primer sequence of quantitative real‐time PCR. [file IID3-14-e70340-s001.docx]

**Supplementary Table S1: Primer sequence of quantitative real-time PCR.**

| **Species** | **Primer name** | **Primer sequence** |
| --- | --- | --- |
| Rat | DUSP1 (Forward) | 5'-TGCTGGAGGAAGGGTGTTTG -3' |
| Rat | DUSP1 (Reverse) | 5'-CGTCCAGCTTCACTCGGTTA -3' |
| Rat | ACSL4 (Forward) | 5'- AGGACTTCCTGGTGCTGTTT -3' |
| Rat | ACSL4 (Reverse) | 5'- CAGGTAGGCTGTGATGGTGA-3' |
| Rat | GAPDH (Forward) | 5'-ATGGCTACAGCAACAGGGT-3' |
| Rat | GAPDH (Reverse) | 5'-TTATGGGGTCTGGGATGG-3' |
